# From didactic explanations to co-design, sequential art and embodied learning: challenges, criticisms and future directions of patient pain education

**DOI:** 10.3389/fpain.2025.1536112

**Published:** 2025-05-09

**Authors:** G. Lorimer Moseley, Amelia Mardon, James Watson, Felicity Braithwaite, Monique V. Wilson, Trevor Barker, James Lawrence, Dianne Sheppard, Michiel F. Reneman, Jennifer Stinson, Cormac G. Ryan

**Affiliations:** ^1^ The Pain Education Team to Advance Learning (PETAL) Collaboration; ^2^ IIMPACT in Health, University of South Australia, Kaurna Country, Adelaide, SA, Australia; ^3^ NICM Health Research Institute, Westmead, NSW, Australia; ^4^ Centre for Rehabilitation, School of Health and Life Sciences, Teesside University, Middlesbrough, United Kingdom; ^5^ Integrated Musculoskeletal Service, North Tees and Hartlepool NHS Foundation Trust, Stockton-on-Tees, United Kingdom; ^6^ Persistent Pain Research Group, Hopwood Centre for Neurobiology, Lifelong Health Theme, South Australia Health and Medical Research Institute (SAHMRI), Kaurna Country, Adelaide, SA, Australia; ^7^ Consumer Advisor, IIMPACT in Health, University of South Australia, Kaurna Country, Adelaide, SA, Australia; ^8^ MedHealth Research, MedHealth, Melbourne, VIC, Australia; ^9^ Monash University Accident Research Centre, Monash University, Clayton, VIC, Australia; ^10^ Department of Rehabilitation Medicine, University Medical Center Groningen, University of Groningen, Groningen, Netherlands; ^11^ Child Health Evaluative Sciences, the Research Institute, The Hospital for Sick Children and Lawrence S. Bloomberg, Faculty of Nursing, The University of Toronto, Toronto, ON, Canada

**Keywords:** chronic pain, back pain, pain education, constructivism, explain pain, virtual reality

## Abstract

Pain Neuroscience Education (PNE) emerged over two decades ago in response to the incoherence between evidence-based pain management strategies, and consumer and clinician understandings of “how pain works”. Many clinical trials have investigated the effects of PNE either as a standalone intervention or embedded within a more complex care package, with mixed results. A range of research methods have been used to explore the inconsistent effects of PNE. Together they (i) identify significant shortcomings and limitations of PNE and (ii) raise the possibility that gaining a broadly scientifically accurate understanding of “how pain works” may be critical for subsequent pain and disability improvements. Both learnings strongly suggest that we need to do better. Extensive research incorporating several interest-holders has led to updated content and language and criticisms of both are addressed. The method of PNE has also been updated, with integration of educational frameworks, teaching strategies and tactics, patient resources and clinical tools that all aim to promote the likelihood that patients will learn key concepts and operationalise them to improve their pain, function and quality of life. Pain Science Education is used to differentiate the new approach from PNE.

## Introduction

About 30 years ago, a new kind of patient pain education emerged. The content of this education focussed on contemporary understandings of neurophysiology of pain in general, and chronic pain in particular. It first emerged as “intensive neurophysiology education” (1–4) and has become best known as “pain neuroscience education”, or PNE (5). Over 35 randomised controlled trials (RCTs) have tested it, against active, sham and no intervention/usual care comparators. About a decade ago, a mixed picture of evidence from these RCTs was emerging; health care professionals (HCPs) were sometimes reporting that PNE is too difficult to do; patients participating in PNE were reporting feeling invalidated. Clinical audit data however, were suggesting that if PNE is effective in changing understanding, then pain and disability reductions would tend to follow. These factors led to substantial changes in content and educational strategy and the development of patient education resources, tools, tactics and guides. The interdisciplinary, international PETAL Collaboration renamed these newer versions “pain science education”, for two reasons: (i) to differentiate, from PNE, patient education that incorporates these developments in content and method, and (ii) to reflect that wider range of scientific knowledge (rather than just “neuroscience”) that is now included. Here we aimed to provide the historical context of PNE, outline the impetus for, and evidence by which, changes in content and delivery strategies have emerged, provide the scientific developments that are driving progress in patient pain education, discuss criticisms of the content, and present a snapshot of the current state of this fast-moving field.

### Historical context: a new kind of pain education emerges as a new intervention

About 30 years ago, rapid advance in the scientific understanding of “how pain works”, facilitated by the seminal discovery of central sensitisation (6) and including pain's protective function, multifactorial nature, dynamic nature over time, and the most effective ways of treating it (7), had created a vast disconnect between scientific and common understanding of the problem of chronic pain. The common understanding remained rooted in a simplistic structural pathology model in which chronic pain was taken to reflect chronic tissue pathology. Evidence-based cognitive-behavioural interventions seemed in contrast to this common understanding. In response to the disconnect, a new educational approach called “intensive neurophysiology education” emerged in 2002 (1–4). The intervention became widely known as “explaining pain” (8), after the primary resource *Explain Pain* (9), and subsequently, “pain neuroscience education” (PNE) (5). This approach is still being widely taught and delivered under that name and in this article we use “PNE” to refer to the original intervention and to differentiate it from more modern approaches to patient pain education.

The impact of this disconnect between common and scientific understanding of pain, could be seen in patient perspectives on pain management programs that focussed not on pain reduction but on living well despite pain (10). The discovery in 1983 of central sensitisation lent weight to the idea that pain reduction was no longer a reasonable expectation. Central sensitisation was demonstrated after peripheral nerve constriction, whereby second order neurones in the spinal cord became upregulated and responsive to non-nociceptive stimuli (6). That discovery led to some seeing chronic pain as an immutable disorder of the spinal cord (11). The original biopsychosocial model (12) also lent weight to this idea—it posited that the impact of pain involves biological, psychological and social influences. Loeser's 1983 adaptation of the biopsychosocial model to the “onion skin” metaphor (13) brought a more pain-specific framework; psychologists were able to re-purpose cognitive behavioural therapies (CBTs) that were established for other diagnoses such as depression, to help patients decrease the impact of pain on their quality of life (14). Guidelines began to include cognitive and behavioural strategies and education about how to use them in the early 2000's (15).

Our consumer-focussed research identified a significant challenge with this shift towards CBT-based pain management. Many people with chronic pain and many health professionals who treated them, found it difficult to reconcile a CBT and self-management based approach with their understanding of “how pain works”—they found the suggestion confusing and invalidating (1, 2, 4). It was an understandable response: if one understands the presence and degree of pain intensity to accurately signal the presence and degree of tissue damage, then interventions that do not directly target those tissues will of course appear to be nonsense. There was a clear need for a new intervention that could provide patients with an understanding of why CBTs and self-management were biologically sensible. PNE therefore, aimed to enable and empower consumers to engage in CBTs and self-management through first explaining why, not just how, to do it. One can readily see the connection between this early mission and the taglines of public-facing modern day public pain education initiatives such as Pain Revolution (https://www.painrevolution.org)—“rethink, re-engage, recover” and Flippin' Pain (https://www.flippinpain.co.uk)—“engage, educate, empower”.

### Clinical trials of PNE have produced mixed results

The earliest randomised controlled trials (RCTs) of PNE compared it to conventional pain education matched for dose, setting and resources, or to an active care comparator, a usual care comparator, or to a waiting list comparator. Those RCTs, in individuals with chronic pain, demonstrated medium to large effects on pain-related neurophysiology knowledge, and small effects on movement-evoked pain, pain-related worry, pain-related self-efficacy, usual pain intensity and pain-related disability (1–4). Since those trials, other trials have been undertaken in various countries, in various settings, and with various diagnostic groups, with the content and format of PNE remaining very consistent. Over 35 RCTs and several meta-analyses on PNE as a stand-alone intervention, and over 50 RCTs of more complex interventions that integrate PNE content, have been undertaken (5, 16–20) (see https://www.petalcollaboration.org/clinical-trials-of-pain-education.html for a list of RCTs). Some studies conclude that PNE as a stand-alone intervention imparts small to medium benefits across a range of outcomes, in a range of chronic pain conditions, in a range of settings and languages e.g., (5), but others report benefit in variables such as knowledge change, catastrphising and fear of pain and (re)injury, but not in pain or disability (19, 21). Opinion pieces advocate for PNE (22–25), but recent commentaries have identified significant challenges and limitations (26–28).

## Real world experience of PNE elucidates challenges and limitations

### Barriers for clinicians

Significant barriers to the implementation of PNE exist. To date, the only major implementation trial in which physiotherapists were trained in PNE (16 h, 8 h online and 8 h face-to-face) or not (29), was a cluster-randomised implementation study that revealed improved pain-related self-efficacy but not better pain or disability outcomes for patients of the PNE-trained physiotherapists. Health professionals have reported significant challenges in delivering PNE such as: limited appointment time; patient-related factors including a lack of perception of education as an intervention in its own right; clinician-specific issues, such as limited confidence in the implementation process (30). Anecdotally, health professionals have told us that (i) some patients do incredibly well but others do not, (ii) they “try the pain talk” or “do explain pain”, but most patients “don’t buy it” or “don’t want it”. Such anecdotes are also borne out in qualitative analysis of primary care practitioner perspectives, which also suggest health professionals attribute difficulties with PNE to too complex content or patient/learner attitude or capacity, but not to a lack of educational expertise or training (31).

Some health professionals consider that the learning objectives of PNE contradict early pioneers of sensory neurophysiology or certain philosophical and semantic principles (32) [see (33) for a pragmatic commentary on the latter]. Others perceive that educating patients about how pain works undermines their own clinical framework or authority, but the largest group understand and accept the merit and intent of pain education and feel they lack the skills and confidence to deliver it effectively (30). Less experienced clinicians have expressed heightened apprehension about using PNE, concerned that unsuccessful implementation could harm therapeutic relationships (30). Although some services, for example many Canadian pediatric pain programs, stipulate education as mandatory before other care commences, health professionals in other jurisdictions (particularly in adult care) have reported that they stopped pain education altogether “because it is too hard and most patients don’t want it” (31). These results are consistent with the apparent ineffectiveness of PNE training for health professionals, at least as it was delivered in one implementation trial (21). Taken together, the available evidence suggests improvements are needed in the content and method of PNE and the training and support of health professionals to implement it.

### Barriers for patients

People living with chronic pain have identified that good pain education is a high care priority, but that they seldom receive it (34). Our real-world data suggest that even “good” education may not be as helpful as we would like. We have investigated this by looking at PNE-related outcomes in clinical practice, outside of research studies. We collected data on knowledge, worry, pain and disability outcomes in a rolling outcome evaluation of over 1,500 consecutive patients, all of whom participated in PNE, with 93% follow-up rate. We used the only assessment for pain knowledge available—the neurophysiology of pain questionnaire (NPQ) (7) and its 12-item revised version (rNPQ) (35). We have previously presented data from 799 of the consumer cohort, all of whom used the original PNQ, and full details of the cohort and the range of diagnoses included, are presented there (36). Two patterns in the outcome evaluation data cast light on the implementation trial outcomes, the qualitative analyses and health professional anecdotes mentioned above.

#### Pattern 1 in the data: after PNE, less than half of all patients demonstrate an understanding of pain biology that aligns with scientific understanding

The vast majority of change in pain-related knowledge, occurred during the phase in which PNE was the dominant component of care. Mean (SD) change during that time was 4.5 (1.6) points, with a range of 0–9 points (total tool range = 0–19 for NPQ and 0–12 for rNPQ; representing a pre-post large effect size ∼1.5) (36). On the basis of extensive previous work (1, 2, 4), we estimated that a score of >10 on the NPQ and >8 on the rNPQ reflected a conceptualisation of pain that is consistent with contemporary pain science. Forty-six percent of patients reached this threshold; all maintained the shift and a further 4% reached the threshold 5 months later. That is, only half of the patients who participated in PNE delivered by experienced pain educators, learnt its key conceptual objectives.

#### Pattern 2 in the data: long-term outcomes are better when learning objectives are achieved early on

Patients completed pain (0–100 Numerical Rating Scale (NRS) of “average pain over that last two days”) and disability [patient-specific functional scale (37)] assessments between 12 and 18 months after their initial appointments. The mean (SD) reduction in usual pain was 36 (19)/100; the mean increase in function was 62 (19)% of the maximum possible score. However, those who achieved the threshold level in NPQ/rNPQ at one month, went on to report a bigger decrease in pain and increase in function than those who did not achieve the threshold level (Table 1).

**Table 1:** A more scientifically based understanding of pain biology early in treatment is associated with a bigger decrease in pain and increase in function 12–18 months later.

Group	Mean (SD) decrease in average pain over two weeks (0–100 NRS)	Mean (SD) increase in function (% of maximum score)
Those who reached NPQ/rNPQ threshold	49 (11)/100	81 (16)%
Those who did not reach NPQ/rNPQ threshold	16 (15)/100	42 (22)%

### Suboptimal patient experiences

The findings from the above outcome evaluation mirror the results of a series of qualitative studies exploring patients’ experience of PNE (one 90 min group session) within clinical settings. The extent of pain reconceptualisation with PNE appears highly variable, for example a positive experience:

“.it also reassured me that I wasn’t going barmy … it [PNE] explained that I’m not. What I am experiencing is real and it explained why, without something necessarily being wrong … things like the sensitivity is a kind of new thing that no one had offered before.” (Participant B post-PNE (38) page 1391)

In contrast, some found the PNE content irrelevant to themselves, their pain and their situation-

“It was just basically stubbing your toe .. I don’t want to know about my toe. I've stubbed my toe, fair enough and I know it last days. But I want to know about why I’ve got the constant pain in my spine. And it just didn't materialise.” (Participant J post-PNE) (39) page 59.

That the content of PNE can lack relevancy, a problem potentially exacerbated by the brief and didactic nature of PNE delivery within a group setting, was a consistent theme across studies (38–40) and points to the need for a stronger consumer voice in guiding the content of pain education. Moreover, even when the content is relevant, and clinical benefits ensue, we have often observed what we call “partial and patchy” reconceptualization: patients describe their pain with some language inconsistent with contemporary pain science:

“Because you assume if you’re in constant pain its damage to the nerves and something you're doing is aggravating it and just what's causing the constant pain rather than it being (reinjured) and it was explained about the with the heightened sensitivity.” (Participant C post-PNE) (39), page 59,

and other language inconsistent with contemporary pain science:

I believe it's the damage to the discs in my spine. (Participant C post-PNE) (39) page 59.

These patterns from real world evaluation data and qualitative research studies show that there is significant room for improvement for PNE in both content and strategy. Participant reflections on a PNE-based complex chronic back pain intervention are corroborative: of all components of the treatment, the educational component was the most difficult; some didn't expect nor want “a pain talk”; some felt invalidated by it; some “couldn’t understand what the health professional was going on about” (41). Although outcome evaluation data do not allow for causal conclusions, they provide a clear hypothesis for testing: when education is successful, the likelihood of reducing pain and disability is high; when it is not, that likelihood is low and may be accompanied by negative therapeutic experiences. It is not unreasonable to propose that reconceptualization of “how pain works” might be a critical determinant of response to care.

## Criticisms of the content of pain science education

The content of Pain Science Education has not escaped criticism. Those criticisms can be grouped into “its content is wrong” and “its delivery is invalidating or dangerous”. These criticisms are rare, but those who hold them are very vocal, primarily through social media channels, or via letters of concern sent to our dissemination and outreach partners, our employers, research funders or the learned societies of which we are members. Respectful and constructive criticism is critical in science, particularly in a field such as ours in which substantial knowledge and translational gaps, inconsistent terminology and field-specific jargon, exist. We take the opportunity here to discuss these criticisms.

### Is the content of modern pain education “wrong”?

It is difficult in science to conclude things to be definitively wrong or right. However, Tables 2–4 present the most common criticisms of the “*Essential Pain Facts*”, derived through the research outlined below under “Potential solutions”.

**Table 2:** Criticisms of the pain science concept “Pain protects us and promotes healing.”

Criticism	Counter-argument
“Nociceptor” is Latin for “harm detector” so pain can’t be protective.	That nociception and pain are different is not contentious in the pain science field, but it remains a common conflation elsewhere. Nociceptors were named over a century ago. Most experiments investigating stimulus-response profiles in human and non-human animals, from Sherrington's field-generating work (42) to current studies, actually do no harm to body tissue. That body of work cannot demonstrate nociceptors as harm detectors, but it does demonstrate that nociceptors code for the intensity of a stimulus [although not necessarily in a linear fashion (43)]. Considering further the link between noxious stimulation and protection, a wide range of physiological responses have been assessed, for example withdrawal, heart rate increase, inflammatory cytokine release, brain activation patterns, coordinated motor responses. Non-physiological assessments include pain report and willingness to participate in another experiment. When multiple trials from multiple individuals are averaged, the intensity of a noxious stimulus relates well to responses, but there is substantial intra-individual and inter-individual variability, even when the setting and stimuli are highly controlled (44). Thus far, all of the responses that have been assessed are consistent with protecting the body from damage either in the moment or when faced with similar threats in the future. Concordantly, most of the pain field has for many decades considered pain a protective response or experience e.g., (45), which is in keeping with an evolutionary lens (46, 47) and has long been an idea central to writings as diverse as Henry James's *Principles of psychology* (48) and William Cullen's *The institutes of medicine* (49). This protective function is captured by the phrase “or potential tissue damage” in the International Association for the Study of Pain (IASP) definition of pain (50).
Pain is a sensation, not a perception or feeling.	The IASP defines pain as a sensory and emotional experience. Our extensive collaborations with consumers have revealed problems caused by multiple meanings of “experience”. It may refer to a state of awareness (“I am *experiencing* pain”), an activity (“I have *experienced* Adelaide at night”) or a state of expertise (“I am an *experienced* clinician”). We have found “perception” can imply blame: “you are perceiving this as pain”—the sufferer feels blamed for feeling a non-painful “thing” as painful. Consumers tell us that “sensation” implies something occurring at the tissue level. “Sensation” is also problematic from a neurophysiological and phenomenological perspective: (i) a diverse array of receptors and neurones are involved in monitoring changes in tissue environment (see (43) for one accessible review); (ii) pain is a unified sensory and emotional experience (50, 51), the dimensions of which may be separately investigated but only under highly controlled experimental conditions (52). For these reasons, Pain Science Education uses the terms experience and feeling, but seldom sensation or perception.
To position pain in consciousness is philosophically problematic	We agree—situating pain as a feeling can be problematic from a philosophical perspective. Positioning *anything* in consciousness is problematic—love, hate, grief and dismay, because consciousness itself remains to be understood. Positioning such things as occurring outside of consciousness is also problematic because we are necessarily aware of pain. We contend that one takes protective action, from turning down the heater or phoning an ambulance, *because they are in pain.*
Pain is not a thing that can promote healing.	The underlying physiology of the effect of injury or inflammation is well understood: when tissue is injured, nociceptors undergo a profound change in their stimulus-response profile, a shift called peripheral sensitisation, which results in primary allodynia and hyperalgesia. The extraordinary process by which this occurs is reviewed in accessible language elsewhere (43). Suffice here to note that the functional impact of peripheral sensitisation is that pain occurs in response to stimuli of intensity well below that which would be dangerous. This sensitivity minimises the likelihood that compromised tissue is exposed to mechanical loads that exceed the tolerance of that tissue.

**Table 3:** Criticisms of the pain science concept “Persisting pain overprotects us and prevents recovery.”

Criticism	Counter-argument
Pain can’t overprotect us because it doesn’t protect us.	The notion that persisting pain becomes “over” protective, reflects two foundational discoveries in our field: central sensitisation (6) and the wide range of similar upregulations that can contribute to, or produce, the same effect: allodynia (pain when pain would not normally be expected) and hyperalgesia (pain more intense than that which would be expected) to a widening arrange of stimulus types. Allodynia provides strong motivation to avoid activities that deliver tissues mechanical loads that keep those tissues strong and healthy, are valued or important for quality of life, and are not in fact dangerous.
There is no such thing as the pain “system”.	We have responded to this criticism elsewhere (33). The term “pain system” is overwhelmingly endorsed by consumers (53, 54), captures the complex, integrated, dynamic, and coordinated nature of pain, and moves away from the previous, and long since undermined by evidence, notion that pain is an isomorphic marker of tissue damage or nociception (55–57). Considering the “pain system” is not a new idea and is widely acceptable (33). Earlier commentary on pain included the ideas of the “action system” and the “somesthetic system” (45).

**Table 4:** Criticism of the pain science concepts “Many factors influence pain” AND “there are many ways to reduce pain and gradually recover.”

Criticism	Counter-argument
These concepts were suggested in order to justify brain-targeted interventions, such as pain education.	This assertion is incorrect. These concepts capture the biopsychosocial nature of pain and predate the emergence of PNE. It is difficult to conceive of an effective pain intervention that does not in some way target the brain. Interventions for chronic pain, such as education and graded motor imagery (58) are based on foundational [e.g., (59)] and clinical (e.g., (60–62) studies. Meta-analyses support their clinical benefit e.g., (17, 63, 64).

### Is the public communication of modern pain science concepts invalidating and dangerous?

We are among those to argue that shifting community norms and consumer expectations around pain care and recovery has the potential to reduce common barriers to participating in guideline-based care and promote better clinical outcomes (30, 65, 66). Our patient partners have also identified that society-wide knowledge translation interventions that target misconceptions about pain and its management, are needed (67). Our recent meta-analyses showed that population beliefs about pain management can be positively shifted in line with guideline-based care (i.e., “staying active”, “avoiding rest”) using contemporary media campaigns (65). However, care must be taken to understand the audience and communicate with them appropriately, so as to avoid the messages being delivered in a poorly contextualised way, leading to misinterpretation, and feelings of invalidation or potentially iatrogenesis (33, 68). We have also demonstrated that approximately half of all public health education programs are unsuccessful for those from marginalised and minoritised communities (ie those who may most need the programs) and that co-developing education programs with the target audience is critical to address their needs (69). We have identified the need for better pain education delivery for disadvantaged groups such as those from ethnically minoritized groups, those with low health literacy, and those who receive their care through an interpreter (68, 70). Relevant here is the wider need across the pain field to better understand the social context of research participants, a need that has triggered a global push to include a minimum equity-relevant data set in all human pain research (71–76).

**Figure 1:**
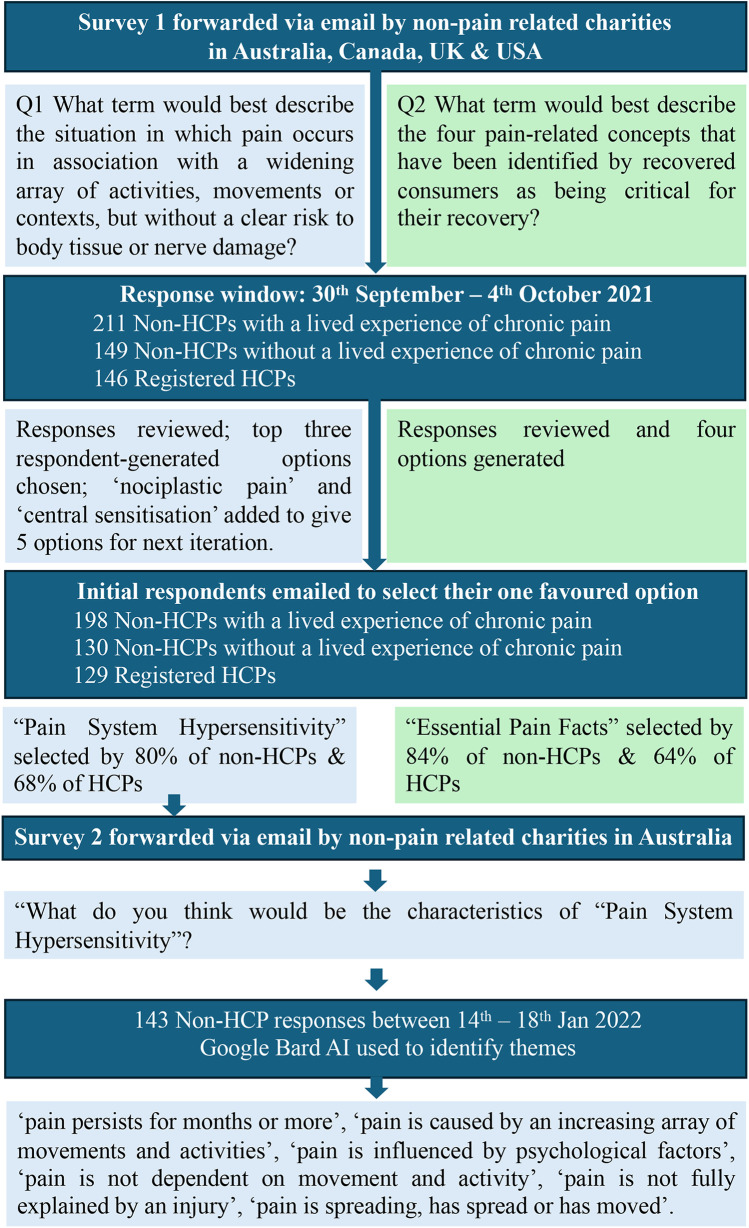
Brief summary of two consumer surveys undertaken by pain revolution, which arrived at the terms “Pain System Hypersensitivity” and “Essential Pain Facts” (survey 1) and validated the former as a suitable proxy for “nociplastic pain” (survey 2). HCP = health care professional; AI = artificial intelligence tool.

Building better pain education involves partnerships with consumers (e.g., members of the public, people with lived experience of pain and their carers/families, and patient reference groups) in the design, planning and delivery of public-facing pain education initiatives and resources [e.g., (77), see also painrevolution.org and flippinpain.co.uk]. Even so, this does not guarantee that all outreach will be acceptable by all people and in all contexts. For example, as part of ongoing efforts to optimise accessibility of Pain Revolution's public facing resources and professional development offerings within its Local Pain Educator, Local Pain Collective, and Rural Outreach Tour initiatives, Pain Revolution regularly consults consumers. It undertook two mini-surveys to arrive at the terms “Essential Pain Facts” and “Pain System Hypersensitivity”. Figure 1 provides a brief summary of these surveys and their results. The themes derived in response to Survey 2 (Figure 1) bear some resemblance to the documented characteristics of nociplastic pain (78).

### Is reconceptualization of how pain works important for subsequent pain and disability improvements?

Recently, three clinical trials of complex multimodal treatments that aimed to shift patients' understanding of the problem of back pain, have shown clinical benefit over and above that offered by their comparator (a sham intervention (79), an open label placebo or usual care (80, 81). Two of those trials were grounded in, and included, PNE (79, 80). They also included planned analyses of whether reconceptualization of the problem contributed to the clinical benefit. The first used a mediation analysis approach and showed that the vast majority of the overall treatment effect of a PNE-grounded intervention [the “Resolve” approach (82)] on pain at post-treatment (global effect of intervention = −0.96 [−1.47 to −0.28]) was mediated by the change in the participants' understanding of their back pain problem, assessed after the PNE component of the program (effect of mediator = −0.96 [−1.47 to −0.64]); the effects were very similar for disability (83). The other clinical trial (80) asked participants before and after treatment about their understanding of the cause(s) of their back pain. They showed that re-attribution of back pain, from tissue-related pathology to psychological stress and brain-related mechanisms, was highly related to clinical improvement (84). These trials of complex interventions suggest that reconceptualization of how pain works seems to be an important determinant of subsequent pain and disability improvements. We contend that these findings are also consistent with the possibility that reconceptualization of “how pain works” might be a critical determinant of response to care in the real world. Efforts to make pain education better seem warranted.

## Potential solutions

### Changing content and method

Numerous scientific studies, which involved hundreds of recovered consumers and health professionals, and dozens of pain, education and learning scientists (28, 30, 33, 38–41, 53, 83, 85–87) have led to significant changes in content and learning objectives. Full coverage of that work is beyond the scope of this review, but in brief: several cohorts of consumers who self-identified as having improved or completely recovered from a chronic pain condition, were interviewed or surveyed about what they learnt or did that lead to their recovery. These data formed the basis of the iterative development of “key learning objectives for pain education” (43, 53) (see https://www.painrevolution.org/painfacts for brief two-page “fact sheets” for each objective). These concepts were prioritised for importance by consumers and cross-checked for accuracy according to published scientific papers, by an interdisciplinary group of active pain researchers, and for acceptability by an interdisciplinary group of practising health and medical professionals. Finally, through collaboration with a health communications firm and more consumer consultation, four key concepts were derived and, through final consumer and clinician surveys, were labelled “*Essential Pain Facts*” (28, 82).

Extensive research has also been directed towards improving how PNE could be implemented more effectively [e.g., (19, 30)]. This has meant drawing on more contemporary approaches to curriculum development and lesson plans [e.g., (43)] and using more contemporary educational frameworks and strategies [see (28)]. Modern pain education takes a constructivist approach to learning, integrates active, constructive, interactive and inventive learning tasks (88), includes a range of teaching “tactics” during educational encounters, and uses novel clinical tools such as sequential art, storytelling, active tasks and virtual-reality mediated embodied experiences. The theoretical frameworks and some strategies have been drawn from education research, where they have been shown to improve learning in over 300 meta-analyses of data from several million participants (89). Other strategies are based on the principles that govern the neurophysiology of learning (43); others are grounded in social cognitive theory (90). It is important to acknowledge that many clinicians have already begun to use contemporary teaching methods to enhance their educational offerings, although up to 40% of health professionals who regularly deliver pain education are unable to name an educational strategy (30).

In 2023, the Pain Education Team to Advance Learning (PETAL) Collaboration was formed. This international, interdisciplinary collaboration of researchers, clinicians and consumers engaged in pain education research and seeking to synergise international efforts, elected to use “Pain Science Education” to describe modern pain education. There were two reasons for this move: (i) to differentiate, from PNE [which is still taught and delivered in its original form (28)], modern patient education that incorporates these developments in content and method, and (ii) to reflect that wider range of scientific knowledge (rather than just “neuroscience”) that is now included.

The intent of making pain education better is clear: maximise learning about how pain works in order to optimise endorsement and adoption of a graded reactivation and self-management approach to *recovery.* The logic is simple: if the didactic “old school” PNE approach is one of the most effective treatments we currently have for chronic pain (91), then much more effective education (i.e., a “stronger dose”) is likely to be more beneficial, and thus deliver better clinical outcomes. Robust clinical trials will support or refute this logic.

### Co-designed resources and sequential art

Co-designed education involves authentic and meaningful engagement with learners (consumers, clinicians and the public) and health professionals throughout the design and development stages, ensuring that tools and resources resonate with their needs, thereby improving acceptability and uptake (92). Pain Science Education draws heavily on co-design methods as well as progress in educational and conceptual change sciences (28). Tools and resources are designed to enrich the learning environment, provide learning opportunities for learners with a wider range of skills and previous educational attainment, and make learning more enjoyable and meaningful—promoting what Hattie and O'Donoghue call the “will” and “thrill” of learning (89). The strategies embedded in these tools are not unique to patient pain education, but have only recently been integrated into it. One such strategy is sequential art, known colloquially as “comics” or “cartoons”, which can be presented in isolation—conveying a specific point— and also as a coherent whole in which certain characters assist to build a longer, cohesive narrative (93). For example, character development of Snoopy and Charlie Brown in Peanuts allows their personality and context to infer complex meanings and inferences (94). Randomised controlled experiments demonstrate that the use of sequential art enhances learning outcomes across several domains (95).

Recent patient-targeted pain education resources for the general public use sequential art (e.g., https://www.tamethebeast.org and flippinpain.co.uk/formula/), for children and youth (96, 97) and for adults with chronic pain (82, 98). The development of these resources involved individual stories to target a specific concept, linking concepts and inferring more complex, nuanced concepts through the development of characters across multiple stories. Extensive consumer involvement during development promotes the relevance, readability and acceptability of the resources, but the international use of the resources means that colloquial language and cultural references, which can offer powerful learning opportunities, are necessarily limited.

We also co-designed a series of brief animated videos in partnership with people living with osteoarthritis to challenge common misconceptions (e.g., osteoarthritis is caused by “wear and tear” of joint cartilage) that reduce participation in guideline-based care. Using Pain Science Education concepts to provide compelling alternative narratives (e.g., “bioplasticity of cartilage”), our pilot data (*n* = 291) showed that a single view trial of these animations led to medium to large reductions in misconceptions in both lay people (with and without OA) and health professionals (99). People with OA also reported reduced fear of movement and an increased intention to exercise, and feedback on the videos was highly positive, with many citing the clear and patient-friendly explanations. The positive reception was unlikely to reflect resonance with pre-held beliefs. To the contrary, the content probably challenged pre-held beliefs: over 70% of consumers reported that it was different from information previously provided to them by health professionals. We do not know whether this reflects poor knowledge or poor education strategy of their previous providers.

The ancient (100) tactic of storytelling can foster deeper engagement with the studied material and has long been thought to improve the extent of learning and retention (101). Storytelling interventions, by altering health-related societal norms, have been shown to impact major public health issues, including sexual health, substance use, hypertension control, and vaccination, across diverse international contexts including culturally diverse communities (102). Storytelling can stimulate and maintain interest in the topic, provide a structure for delivery and remembering the material, and improve the connection between the educator and the learner (103, 104). Examples in the pain field include the children's comic book “*Medikidz Explain Chronic Pain: What's Up with Moira's Grandad*” (105) and “*Painful Yarns. Metaphors and stories to help understand the biology of chronic pain*” (106)*.* The former addresses the challenges that older people with chronic pain experience when trying to explain to their grandchildren why they are sometimes unable to play with them because of their condition. The latter consists of short stories used as metaphors for pain science concepts; a small clinical trial suggested it improved understanding of pain biology (107). Our patient partners have also highlighted the potential of patient testimonial storytelling: “*Storytelling is powerful. There is nothing like someone else's experience with a good outcome—it shows that it can work.*” Supplementary material 1 [(67), p. 6].

### VR-enhanced pain education and modern pain education tactics

One tool that has enhanced educational outcomes in mainstream education is virtual reality (VR)-mediated embodied learning experiences. A meta-analysis of 43 trials, undertaken in several education settings, reported medium to large effect sizes on a range of learning outcomes, including cognitive, intellectual, motor and behavioural outcomes (108). VR-enhanced education creates experiences within a virtual environment, usually via head-mounted goggles, that target specific learning objectives. Using VR to enhance health education has been explored in women undergoing breast cancer (109) or cardiac (110) surgery. VR is not new to the chronic pain space (111), and others have delivered the didactic “presentation-based’ PNE using VR goggles (112, 113), reporting positive results. Using VR to deliver content in a similar way to how it might be delivered face-to-face or online seems a missed opportunity—the learning potential created by VR is likely to come from generating compelling and memorable experiences and active learning activities.

We have assisted one VR-based platform in its objective to generate experiences and activities, to achieve deep learning of PSE's learning objectives. Three independent research groups have undertaken preliminary investigation: Skidmore et al. (114) found that the Reality Health VR-enhanced pain education platform (Reality Health, Sydney, Australia) is easy for health professionals to use; provides credibility for using education as an intervention; provides users with safe experiences that reinforce and rapidly consolidate PSE's learning objectives. Mardon et al. (115) found it to be feasible, acceptable and safe for use with war Veterans with and without post-traumatic stress, and health professionals who treat them: key themes were novelty, compelling learning experiences, enjoyable, easy to use, and made complex concepts easy to understand. Kennedy et al. (116) evaluated the platform with patients on a waiting list for specialist pain services in Australia: 28 participants, 11% identifying as Australian Aboriginal origin and 9 as being born overseas, rated highly its ease of use (4.6/5), enjoyment (4.7/5), and their desire to use it again (4.3/5); 23 reported improvement with physical activities and mood, and 19 reported improvement in pain levels after the VR-based program. Clinical audit data appear promising: 315 long-term injured workers completed a VR-enhanced pain education program, showing substantial pre-post reductions in pain, pain interference and self-efficacy (full outcome evaluation data are presented with permission at https://www.petalcollaboration.org). Whether or not VR-enhanced PSE improves clinical outcomes more effectively than PSE without VR is yet to be investigated in a clinical trial.

With or without access to VR-enhanced pain education, a range of tactics can be employed by pain educators to enhance deep learning of PSE's learning objectives (82, 98). Using a modified ICAP conceptual change framework (88, 117), tasks that foster deep learning, from “active”, to “constructive” and “interactive/inventive” tasks, should improve pain education outcomes, just as they have been shown to do in conventional educational contexts. The RECLAIMA acronym (Table 5) can guide clinicians towards simple tactics that they can use “in the moment” to push learners towards deeper learning, and the SALAD task (28) can be used to integrate constructive and inventive strategies into reading, video-based or audio-based “homework”.

**Table 5:** The RECLAIMA tactics list. Letters in parentheses (A), (C), (I), denote the level of learning involved according to a modified version of the ICAP Framework (88)—Active (A), Constructive (C) or Interactive/Inventive (I).

	Definition	Description/example prompts and exercises
R	Repeat, recall (A), reflect (C)	Repeating key phrases word for word; asking the learner to recall a previous concept or learning experience; asking the learner to reflect on what a given concept may mean for them, or to scan for feelings that may be related to the new content
E	Explain to another (A,C,I)	Explain this back to me (also known as “talk back”); explain it to another patient, your partner, your pet
C	Compare and contrast (C,I)	How does that concept compare, contrast or contradict what you previously thought or have heard elsewhere
L	Land? (A)	How did that land with you? Look for “knowledge shields”—behavioural cues that a learner is disengaging from the content (see (82, 98) for more detail)
A	Analogy or metaphor (I)	Can you think of an analogy or metaphor to bring understanding of that concept to another person currently without your understanding of it?
I	Investigate more? (A)	Are you curious about anything we discussed? Would you like to investigate anything more?
M	Mode switch (I)	Can you draw what we just discussed? Can you describe this image in words?
A	Action (I)	What could you do differently on the basis of what we have just discussed? What will you do differently on the basis of what we have just discussed? How will you hold yourself accountable to making that change?

## Conclusion and summary

We aimed to provide an account of pain education focussed on imparting scientifically accurate understanding of “how pain works”, its emergence over 20 years ago and its substantial adaptation and growth since then. RCTs consistently show improvements in cognitive variables such as knowledge, self-efficacy and fear, but evidence around pain and disability improvements is mixed. A range of methods have been used to demonstrate that reconceptualization of “how pain works” may be a key determinant of subsequent clinical improvement. Critically, challenges to the implementation of early iterations clearly identified the need for more relevant content and better educational strategy. These content and strategy changes have led to what is now termed Pain Science Education, which targets specific learning objectives broadly covered by the *Essential Pain Facts*, and includes a range of strategies and clinical tools and resources.

Where to from here? We contend that the health education field has great scope for improvement and that there is a pressing obligation for us to make pain education better. Our research groups are prioritising co-design with consumers and end users. We are also exploring methods to push modern understanding to the wider public, for example via Pain Revolution (https://www.painrevolution.org), Flippin' Pain (https://www.flippinpain.co.uk) and the EQUiPP Project (https://www.equipp.org.au), which is employing a “micro-community” precision co-design approach to formulating general public pain messaging strategies. Further priorities include increasing knowledge and skills among health professionals across the health system and development of resources to assist health professionals fast track the changes in understanding that seem to most closely relate to subsequent improvement and often recovery.
